# Associations between circadian misalignment and telomere length in BD: an actigraphy study

**DOI:** 10.1186/s40345-022-00260-w

**Published:** 2022-05-27

**Authors:** Luana Spano, Vincent Hennion, Cynthia Marie-Claire, Frank Bellivier, Jan Scott, Bruno Etain

**Affiliations:** 1 INSERM UMR-S 1144, Optimisation Thérapeutique en Neurospsychopharmacologie, OTeN, Université de Paris, 75006 Paris, France; 2 Université de Paris, Paris, France; 3 DMU Neurosciences, Département de Psychiatrie Et de Médecine Addictologique, AP-HP.Nord, GH Saint-Louis-Lariboisière-F. Widal, Paris, France; 4 Translational and Clinical Research Institute, Newcastle University, Newcastle upon Tyne, UK; 5 Département de Psychiatrie et de Médecine Addictologique, Centre Expert Troubles Bipolaires, Hôpital Fernand Widal, 200, rue du Faubourg Saint Denis, 75010 Paris Cedex, France

**Keywords:** Bipolar disorder, Telomere, Circadian, Actigraphy, Morningness, Eveningness

## Abstract

**Background:**

Life expectancy is significantly decreased in bipolar disorder (BD). This is associated with accelerated cellular aging which can be estimated by telomere length (TL). However, specific determinants of shorter TL in BD are under-explored. This study examines whether circadian misalignment (i.e. mismatch between preferred and actual phase of circadian activity rhythms) is associated with shorter TL in BD.

**Methods:**

Euthymic individuals with BD (n = 101) undertook 21 consecutive days of actigraphy recording and completed the Composite Scale of Morningness (CSM) to assess phase preference for activities (chronotype). Polymerase chain reaction was used to measure TL in blood. Cluster analysis identified circadian aligned/misaligned subgroups as defined by preferred (CSM score) and actual phases of activity (actigraphically determined onset of active and inactive periods). We tested for any associations between TL and clusters, with adjustments for between-cluster differences in socio-demographic and illness factors.

**Results:**

We identified three clusters: an "Aligned Morning" cluster (n = 31) with preferred and actual timing of activity in the morning, an "Aligned Evening" cluster (n = 37) with preferred and actual timing of activity in the evening and a "Misaligned" cluster (n = 32) with an evening chronotype, but an earlier objective onset of active periods. After adjustment for confounders, we found that TL was significantly associated with circadian misalignment and older age.

**Conclusions:**

Circadian misalignment may partly explain shorter TL in BD and could contribute to accelerated aging in these individuals.

## Introduction

Life expectancy in individuals with BD is reduced by about 10–15 years compared with the general population. While suicide risk contributes significantly to the excess of premature mortality in BD, the main causes of premature mortality are natural and related to multiple comorbid somatic conditions (Kessing et al. 2015a, 2015b), especially age-related non-communicable diseases, such as cardiovascular and metabolic disorders. These findings have led to the hypothesis of accelerated cellular aging in BD (Rizzo 2014), which can be estimated by telomere length (TL). Telomeres are DNA repeat sequences, located at the end of chromosomes, and protect chromosomes against degradation and fusion (Aubert and Lansdorp 2008). The length of telomeres decreases with age, because of the incomplete DNA replication at each cell division, making it a marker of cellular aging (Blackburn et al. 2015). A meta-analysis (10 studies—1,130 participants) demonstrated a significant reduction in TL in individuals with BD as compared to controls (Hedges’ g =  − 0.54 (− 0.84;-0.28), p < 0.001) (Huang et al. 2018).

The determinants of telomere shortening in BD are poorly understood. Several hypotheses have been proposed, such as genetic vulnerability, exposure to childhood adversity and maltreatment, illness progression (e.g., TL may decrease after each new episode), or the over-representation in individuals with BD of other factors associated with shorter TL (e.g. obesity or smoking) (Aas et al. 2019; Birkenaes et al. 2021; Bortolato et al. 2017; Gielen et al. 2018; Li et al. 2017; Martinsson et al. 2013; Powell et al. 2018). Interestingly, it is suggested that lithium, which is widely used as a mood stabilizer in BD, may be "protective" against telomere shortening (Coutts et al. 2019; Martinsson et al. 2013; Pisanu et al. 2020; Squassina et al. 2016). An additional, but unexplored, hypothesis can be proposed: circadian disturbances that are core features of BD might be associated with telomere shortening.

Evidence suggests that there are negative consequences for both health and cellular aging when the circadian system is disrupted or challenged (Banks et al. 2016) and that telomere shortening might be caused by disruptions in the biological clock (Kagawa 2012). For example, in mice and in drosophila, laboratory-induced circadian desynchronization (i.e. non-24-h dark/light cycles) can lead to senescence, telomere shortening and decreased longevity (Boomgarden et al. 2019; Grosbellet et al. 2015; Inokawa et al. 2020). In human studies, individuals engaged in night-work or shift-work (e.g. nurses, or flight personnel) may experience a higher risk of all-cause mortality (mostly from cardiovascular diseases, and possibly from cancers) (Ballard et al. 2000; Su et al. 2021) and greater telomere attrition (Carugno et al. 2019; Pavanello et al. 2019; Samulin Erdem et al. 2017). Since both TL shortening and circadian disturbances are common in individuals with BD (Huang et al. 2018; Murray et al. 2020), their potential co-association may be of particular interest. To date, there is limited direct evidence that TL shortening is associated with circadian disturbances in BD, although the Netherlands Study of Depression and Anxiety reported associations between TL and both preferred phase (evening-type, i.e. a preference for engaging in activities later in the day) and actual phase (i.e. later sleep-onset time) (Wynchank et al. 2019). These findings made us question whether TL may be associated with particular types of circadian disturbances such as "circadian misalignment".

This term "circadian misalignment" has been propose to describe *"a variety of circumstances, such as inappropriately timed sleep and wake, misalignment of sleep/wake with feeding rhythms, or misaligned central and peripheral rhythms"* (Baron and Reid 2014). More generally, circadian misalignment may represent a mismatch between endogenous circadian rhythms (i.e., associated with circadian phase preference) and effective behaviors (e.g., activity patterns imposed by social and work schedules or other routines). A recent meta-analysis identified that individuals with BD are typically characterized by an evening chronotype (Meyrel et al. 2021), so it is possible that they may experience a mismatch between their preferred phase of activity or chronotype (for instance assessed by morningness-eveningness questionnaires) and their actual phase of activity (for instance measured by actigraphy) due to work schedule or other forced routines.

Given the evidence we reviewed, we hypothesize that (1) some individuals with BD will demonstrate a mismatch between their preferred and actual phases of activity (hereafter referred as circadian misalignment) and that (2) this circadian misalignment will be associated with shorter TL. For this purpose, we examined data about TL and circadian alignment and misalignment (based on preferred phase of activity as measured by a self-report of morningness-eveningness and actigraphy estimates of timing for activity/inactivity) that was collected from a large clinical sample of euthymic individuals with BD.

## Materials and methods

### Participants

Participants were recruited from a university-affiliated psychiatric department in Paris (France). Written informed consent was obtained from all participants. This research protocol (Clinical Trials Number NCT02627404) was approved by the French medical ethics committee (Comité de Protection des Personnes (CPP)–IDRCB2008_AO1465_50 VI–Pitié-Salpêtrière 118–08) and carried out according to the approved guidelines.

Inclusion criteria were: age ≥ 18 years, a DSM-IV diagnosis of BD type I or II according to the SCID (Structured Clinical Interview for DSM Disorders) (First et al. 1995), being euthymic for ≥ 3 months, with a current MADRS score < 8 (Montgomery and Asberg Depression Rating Scale) (Montgomery and Åsberg 1979), and a current YMRS score < 8 (Young Mania Rating Scale) (Young et al. 1978); and willing and able to give written informed consent.

Exclusion criteria were: being unable to undertake actigraphy monitoring for 21 consecutive days, current alcohol or substance misuse problem (excluding current tobacco use), inpatient treatment and/or modification of mood stabilizer regime in the 3 months prior to assessment, employment involves nightwork or shiftwork, recent trans-meridian travel, current diagnosis for a comorbid neurological or sleep disorder (narcolepsy, obstructive sleep apnea, restless leg syndrome), being prescribed a non-psychotropic medication that can alter sleep and circadian rhythms (such as cortisone), and/or any other mental or physical health problem that contradicted participation in the study.

### Clinical measures

At baseline, we collected information about participants’ age, sex, Body Mass Index (BMI), tobacco use (current, past or never), past lifetime history of alcohol misuse, MADRS and YMRS scores, age at onset of BD (age of first full-threshold mood episode that met DSM-IV criteria), duration of illness, number of BD episodes, and currently prescribed medications. The latter were classified as mood stabilizers (lithium, anticonvulsants, atypical antipsychotics), antidepressants and benzodiazepines.

Participants completed the French version of the Composite Scale of Morningness (CSM) (Smith et al. 1989) which is a 13 items self-report that assesses preferred sleep/wake times, peak cognitive performance and morning affect. The total score can range from 13 to 55. A previous French validation study indicated that a CSM score > 44 is indicative of morningness, whilst a score < 32 is indicative of eveningness (scores between 32–44 are categorized as intermediate) (Caci et al. 1999).

### Actigraphy recording

Participants wore an actiwatch (the CamNtech AW-7) continuously on the non-dominant wrist for 21 consecutive days. For this study, an epoch of 1 min and an “average” sensitivity threshold was chosen. Participants were shown how to press a button on the device when they went to bed at night and when they got up in the morning (as this enabled the investigators to determine rest periods). Actiwatch Sleep and Activity software program (V7.28) was used to estimate a range of actigraphy metrics. For the purposes of this study, we extracted data on two parameters: M10 onset (representing the clock time for the onset of the most active 10-h period) and L5 onset (onset of the least active 5-h period). Our aim was to compare the above actigraphy estimates with scores from self-rated CSM to identify individuals with circadian alignment (i.e., evidence of concordance between self- and objective estimates of most and least active time periods) or misalignment (i.e., evidence of discordance between estimates).

### Telomere length measurements

Genomic DNA was isolated from peripheral blood using an automated Maxwell 16 DNA Purification Instrument (Promega). TL was measured using real-time quantitative polymerase chain reaction (PCR) in a 10 μL of final reaction volume containing 25 ng of DNA, 5 μL of 2 × SSoAdvanced Universal SYBR Green Supermix (biorad) and 300 nmol/L of each following primers: TL-F (TL 5’-CGG-TTT-GTT-TGG-GTT-TGG-GTT-TGG-GTT-TGG-GTT-TGG-GTT-3’); TL-R (5’-GGC-TTG-CCT-TAC-CCT-TAC-CCT-TAC-CCT-TAC-CCT-TAC-CCT-3’) for telomere and beta_hemoglobin-F (5’-GCT-TCT-GAC-ACA-ACT-GTG-TTC-ACT-AGC-3’); beta_hemoglobin-R (5’-CAC-CAA-CTT-CAT-CCA-CGT-3’) for beta-hemoglobin as previously described (Tyrka et al. 2015, 2016).

Thermocycling conditions were as follow: initial heating step of 98 °C for 3 min followed by 40 cycles of 98 °C for 15 s and 60 °C for 30 s. All reactions were performed using three technical replicates and data were acquired using a CFX384 Touch Real-Time PCR Detection System (Biorad). A relative TL was estimated using beta-hemoglobin gene as a single-copy gene and the delta delta Cq calculation method (Pfaffl 2001).

### Statistical analysis

Statistical analyses were performed using SPSS (version 26) and significance was set at p < 0.05.

Sample characteristics are described using means (with standard deviations), medians (with interquartile ranges; IQR) or counts and percentages as appropriate.

First, we used partial correlations (controlling for age) to examine the associations between TL, CSM score, M10 onset and L5 onset.

Second, to identify which individuals had circadian alignment or misalignment, we entered the CSM total score, M10 onset and L5 onset times into a two-step cluster analysis. This analysis allowed us to identify the optimal number of clusters (of aligned and misaligned subgroups). The procedure uses a distance measure (reflecting the (dis)similarity between two observations) based on the log-likelihood, and a clustering criterion based on the Schwarz's Bayesian Criterion (BIC). The characteristics of the identified clusters were then compared using Kruskal–Wallis tests (for continuous variables) and Chi^2^ or Fisher’s exact tests (for categorical variables).

Third, we used a Generalized Linear Modelling (GLM) to examine any association between TL and clusters, while adjusting for potential confounders. TL was used as the dependent variable. Independent variables were clusters (used as an ordinal variable), any variable that would differ between clusters (selected according to p values < 0.10 in the univariable analyses) and factors that might be associated with shorter TL (such as BMI and current tobacco use). The analyses were also adjusted for age, sex, BD type and currently prescribed medications (represented by three separate variables: number of mood stabilizers, antidepressants and benzodiazepines).

## Results

The sample comprised 101 individuals with BD. As shown in Table 1, the sample median age was about 43 (IQR 34–54), most individuals were female (61.4%), and nearly 75% had a diagnosis of BD-I. Most participants were being prescribed  ≥ 1 mood stabilizer (n = 95) with lithium being prescribed most often (65%), followed by anticonvulsants (47%) and atypical antipsychotics (32%). Median time of M10 onset was 10:00 h in the morning and median time of L5 onset was 01:00 h in the morning. The median CSM total score was 36 (IQR 30–42). Most participants had an intermediate chronotype (52.5%) while about 30% were evening types and about 18% were morning types. The sample median relative TL was 2.9 (IQR 2.3–3.8).

**Table 1:** Socio-demographic and clinical characteristics of the sample (N = 101)

Variables	N	%	Median	IQR
Sex (females)	62	61.4%		
Age			42.6	33.8–54.2
BD type 1	75	74.3%		
Age at onset			24	20–30
Duration of BD			15	10–25
Number of episodes			6	4–9
Number of mood stabilizers			1	1–2
Lithium	66	65.3%		
Anticonvulsants	48	47.5%		
Antipsychotics	32	31.7%		
Antidepressants	29	28.7%		
Benzodiazepines	11	10.9%		
Body Mass Index			24.5	22.5–27.8
Sleep apnea risk	19	18.8%		
Tobacco current smoker	48	47.5%		
Past lifetime alcohol misuse	31	30.7%		
MADRS			2	0–4
YMRS			0	0–1
M10 onset (hours)			10:00	8:00–11:00
L5 onset (hours)			1:00	0:00–2:00
CSMa total score			36	30–42
Chronotype categories				
Morning	18	17.8%		
Intermediate	53	52 5%		
Evening	30	29.7%		
Telomere length			2.9	2.3–3.8

IQR: interquartile, BD: Bipolar Disorder, MADRS: Montgomery Asberg Depression Rating Scale, YMRS: Young Mania Rating Scale, CSM: Composite Scale of Morningness

^a^A lower CSM scores is indicative of an evening type (range: 13–55)

As shown in Table 2, the cluster analysis generated 3 subgroups. Given the CSM and actigraphy estimates, we labelled the clusters as: Aligned Morning, Aligned Evening and Misaligned. Individuals in the Aligned Morning cluster (n = 31) tend to have a morning chronotype and have an early start time for active and inactive periods (respectively 08:00 in the morning and midnight). Individuals in the Aligned Evening cluster (n = 37) tend to have an evening chronotype and a later start time for active and inactive periods (respectively 11:00 in the morning and 02:00 in the morning). Misaligned individuals (n = 32) were characterized by a tendency to an evening chronotype, but a moderately early start of active and inactive period (respectively 09:00 in the morning and 01:00 in the morning).

**Table 2:** Comparisons between clusters for Composite Scale of Morningness total score, M10 onset and L5 onset values

Variables	Aligned Morning (AM) n = 31	Aligned Evening (AE) n = 37	Misaligned (MisA) n = 32	p values	Group comparisons
CSM^a^	42 (39–46)	34 (27–38)	32 (30–35)	10^–10^	AM > AE = MisA
M10 onset^b^	8:00 (7:00–9:00)	11:00 (10:00–12:00)	9:00 (9:00–10:00)	10^–15^	AM < MisA < AE
L5 onset^c^	0:00 (0:00–1:00)	2:00 (2:00–2:30)	1:00 (1:00–1:00)	10^–16^	AM < MisA < AE

CSM: Composite Scale of Morningness. Reported values represent medians and Interquartile ranges

^a^A lower CSM scores is indicative of an evening type (range: 13–55)

^b^A higher M10onset is indicative of a later period of activity during the day

^c^A higher L5onset is indicative of a later period of rest during the night

Table 3 shows the socio-demographic and clinical characteristics of each cluster. Three variables significantly differentiated between the clusters: age at inclusion (p = 0.002), duration of BD (P = 0.006) and tobacco use (p = 0.03).

**Table 3:** Comparisons between clusters for socio-demographic and clinical characteristics

Variables	Aligned Morning n = 31	Misaligned n = 32	Aligned Evening n = 37	p values	Group comparisons
Sex (females)	58%	72%	57%	0.39	
Age	54 (38–62)	44 (35–59)	38 (31–43)	**0.002**	**AM = MisA > AE**
BD type 1	77%	66%	78%	0.42	
Age at onset	26 (20–33)	24 (20–30)	21 (19–30)	0.49	
Duration of BD	23 (12–31)	17 (12–25)	12 (6–17)	**0.0006**	**AM = MisA > AE**
Number of episodes	8 (6–10)	10 (6–21)	8 (4–12)	0.27	
Lithium	71%	62%	62%	0.70	
Anticonvulsants	52%	53%	40%	0.51	
Antipsychotics	29%	34%	32%	0.91	
Antidepressants	32%	41%	16%	0.08	
Benzodiazepines	19%	6%	5%	0.15	
Body Mass Index	25 (23–28)	25 (22–26)	23 (22–28)	0.39	
Sleep apnea risk	10%	25%	22%	0.26	
Current tobacco use	32%	47%	65%	**0.03**	**AM < MisA < AE**
Past alcohol misuse	30%	29%	36%	0.79	
MADRS	1 (0–3)	2 (1–6)	2 (0–4)	0.07	

Categorical variables are reported as percentage. Continuous variables are reported as median and interquartile

BD: Bipolar Disorder, MADRS: Montgomery Asberg Depression Rating Scale

As shown in Table 4, the multivariable analysis (Table 4) showed that TL was shorter in individuals being Misaligned and in older individuals. The effect of age and circadian misalignment were independent from each other (respectively, p = 0.04 and p = 0.02).

**Table 4:** Multivariable analysis of telomere length according to clusters of circadian misalignment and confounders

Variables	Beta	SE	Wald Chi-Square	df	p value
Aligned Morning	0.009	0.455	< 0.001	1	0.98
Misaligned	− 0.547	0.237	5.335	1	**0.02**
Aligned Evening (reference)	–	–	–	–	–
Age	− 0.029	0.014	4.127	1	**0.04**
Female	0.289	0.216	1.786	1	0.18
BD type 1	0.094	0.266	0.123	1	0.73
Duration of BD	− 0.007	0.016	0.194	1	0.66
MADRS	0.076	0.056	1.836	1	0.17
BMI	− 0.002	0.022	0.009	1	0.92
Current tobacco use	0.46	0.243	3.579	1	0.06
Number of mood stabilizers	− 0.038	0.158	0.058	1	0.81
Antidepressants	− 0.154	0.252	0.372	1	0.54
Benzodiazepines	− 0.682	0.443	2.364	1	0.12
Intercept	4.227	0.791	28.555	1	< 0.001

SE: Standard Error, df: degree of freedom, BD: Bipolar Disorder, MADRS: Montgomery Asberg Depression Rating Scale, BMI Body Mass Index

In addition, we performed a more parsimonious model that included only variables retained by the univariable analyses with p values < 0.05, i.e. circadian misalignment, age, duration of BD and current tobacco use. We found similar results and observed independent associations between TL and age (p = 0.044) and between TL and circadian misalignment (p = 0.025) (model not shown in details, available on request). In this model, the associations between TL and current tobacco use (p = 0.09) and between TL and duration of BD (p = 0.68) were not significant.

## Discussion

Previous research has demonstrated shorter TL in BD compared with healthy controls (Huang et al. 2018). To our knowledge, this is the first study to suggest that telomeres are shorter in a subset of individuals with BD who demonstrated circadian misalignment, i.e., who have a mismatch between their preferred and actual phase of greatest activity. Furthermore, this association was independent of the association between TL and age and remained statistically significant after adjusting analyses for potential confounders (as identified in previous research).

This study focused on a ‘created’ variable called circadian misalignment, which was based on the discordance between objectively assessed timing of activity and self-reported preference for greater activity in the morning or evening (also referred to as chronotype). The current study identified that around one third of individuals with BD experience circadian misalignment. Interestingly, this type of misalignment is typically seen in adolescents (and is sometimes referred to as "social jetlag") (Henderson et al. 2019), but also in other sub-populations, such as shift-workers (Roenneberg et al. 2019). The explanation of the observed pattern is usually presented as follows. Across the lifespan, chronotype typically shifts, from greater eveningness during adolescence to greater morningness in older adults (Randler 2016). When allowed to follow their preferred patterns (during weekends or vacations, etc.), individuals with an evening chronotype typically have later pressure for sleep, later bedtime, later wake time, and a slower dissipation of sleep in the morning. Conversely, during entrainment (e.g., forced regular weekly routines), individuals with an evening chronotype experience more sleep deprivation, leading to a chronic sleep debt compared with individuals with a morning chronotype (Taillard et al. 2021). Greater eveningness may therefore increase the risk of circadian misalignment between the endogenous biological clocks and the timing of regular weekday activities (with required onsets and offsets of certain activities).

Several hypotheses may explain our finding that this type of circadian misalignment may be associated with shorter TL. For example, misaligned individuals are more prone to sleep loss and sleep disturbances that may shorten telomeres (Tempaku et al. 2015). Also, evening types reported higher levels of tobacco and alcohol use (Evans and Norbury 2021), which some researchers suggest may be a strategy employed to counterbalance wake effort in the morning and/or to promote sleep in the evening (Taillard et al. 2021). Both tobacco and alcohol use have been associated with shorter TL in meta-analyses (Astuti et al. 2017; Navarro-Mateu et al. 2021). Moreover, circadian misalignment may lead to circadian disturbances in cortisol secretion rhythms (Huang et al. 2021) and, in turn, cortisol reactivity has been associated with telomere shortening (Jiang et al. 2019).

This study has several strengths, including a relatively large size of the sample for a study of TL in BD (most previous studies included < 40 cases), and our analyses considered several potential confounders. Nevertheless, the key limitations should be discussed. First, the CSM is a self-report measure that is at risk of individual biases. Secondly, our objective measures of activity (e.g., M10 onset) were not adapted to take account of the participants' employment status or other "zeigtgebers", such as regular routines involving child care responsibilities, etc. Thirdly, and most important, there is no consensus definition of circadian misalignment and we acknowledge that other approaches (such as those that rely on differences in weekday and weekend patterns of activity) might yield different findings (Roenneberg et al. 2019). Fourthly, whilst we had detailed information about currently prescribed medications, our analyses did not consider dose or duration of treatment exposure, nor was it feasible to explore putative links to each mood stabilizer (such as lithium), or to specific combinations of mood stabilizers. This is potentially important as, for example, duration of treatment, especially of lithium, may be associated with a longer TL (Squassina et al. 2016). Finally, the original cross-sectional study was not specifically designed to evaluate circadian misalignment or any longitudinal association with telomere shortening. Also, we did not have an a priori statistical power analyses applicable to this specific project and, as this was an exploratory study, we have not applied any correction for multiple testing. All these issues mean our findings need replication.

If our findings are confirmed, this study may open avenues for a better understanding of the mechanisms leading to telomere shortening in BD and consideration of strategies to slow-down the phenomenon. Targeting some modifiable behavioral risk factors that have been associated with shorter TL (smoking, physical inactivity, obesity, poor diet, substance abuse) may lead to a gain in life expectancy of three years at age 50 years in BD (Dregan et al. 2020). Our study offers an additional target for intervention in BD that is the mismatch between preferred and actual circadian phase of activity. Chronotype (i.e., an evening preference) is suggested to be moderately modifiable (Leocadio-Miguel et al. 2021). Nevertheless, some interventions targeting light exposure, fixed meals times, caffeine intake and exercise may induce a phase advance in individuals with late chronotypes, (Facer-Childs et al. 2019; Thomas et al. 2020). Such an effect has not been explored yet in a population with BD. Additionally, one simple way to reduce circadian misalignment would be to encourage individuals to adjust their work schedule or other regular routines to match more closely the individual's chronotype. This might be done through simple self-help techniques, the use of digital apps or more formal chronotherapies (Gottlieb et al. 2019). In this context, we also note that lithium might prevent telomere attrition, and that its efficacy in BD may partly be mediated by its stabilizing effect on circadian rhythms (Xu et al. 2021). More research is therefore needed to test whether any of the potential manipulations of the biological clock and/or attempts at creating a better fit between actual and preferred timing of activity would lead to some benefit for outcomes in BD, especially in terms of cellular aging. Future research that attempts to integrate biological and psychological approaches might examine whether putative chronotherapy interventions are associated with less telomere attrition that control interventions.

In conclusion, we suggested that TL was shorter in a subpopulation of individuals with BD who displayed circadian misalignment, even after adjustment for chronological age and other potential confounders. If findings are replicated, it may encourage routine clinical measurements of preferred activity phase to identify individuals with circadian misalignment as they may be at greater risk of telomere attrition. Another potential outcome of this study is that it may open up new avenues of research such as the exploration of whether interventions targeted at any circadian mismatch in individuals with BD not only improve the clinical course and outcome, but reduce the risk of premature cellular aging.

## Data Availability

Due to ethical and legal restrictions, data involving clinical participants cannot be made publicly available. All relevant data are available upon request to the authors for researchers who meet the criteria for access to confidential data.
